# DDX21 Is a Potential Biomarker for Predicting Recurrence and Prognosis in Hepatocellular Carcinoma

**DOI:** 10.1155/ancp/1018820

**Published:** 2025-01-18

**Authors:** Chengjie Ji, Qing Zhong, Huilan Su, Xiaoli Xue, Renxiang Yang, Na Li

**Affiliations:** ^1^School of Public Health, Chengdu Medical College, Chengdu, Sichuan, China; ^2^Department of Laboratory Medicine, The People's Hospital of Jianyang City, Chengdu, Sichuan, China; ^3^Department of Anesthesiology, The People's Hospital of Jianyang, Chengdu, Sichuan, China; ^4^Department of Laboratory Medicine, Yingshan County People's Hospital, Nanchong, Sichuan, China

**Keywords:** biomarker, DDX21, hepatocellular carcinoma, prognosis, recurrence

## Abstract

DEAD-box helicase 21 (DDX21) is a conserved Asp-Glu-Ala-Asp (DEAD) box RNA helicase with multiple functions that is involved in various cellular processes and diseases. However, the role of DDX21 in the recurrence and prognosis of hepatocellular carcinoma (HCC) patients remains unknown. In the current study, we examined the protein expression of DDX21 in HCC tissues through immunohistochemical staining and analyzed the correlation between DDX21 protein expression and clinical outcome via Kaplan–Meier survival analysis. The Cox proportional hazards regression model was used to assess the interrelationships between the outcome and variable over time. Our results showed that increased expression of DDX21 protein was observed in HCC tissues compared with paracancerous tissues and was associated with advanced BCLC stage. Recurrent HCC patients had higher levels of DDX21 protein than nonrecurrent cases. Notably, DDX21 was an independent risk factor for predicting worse overall survival and recurrence-free survival in HCC patients. Furthermore, lack of DDX21 abated the growth and mobility of Hep3B cells. Taken together, our data highlight the clinical significance of DDX21 in the recurrence and prognosis of HCC patients and indicate that targeting DDX21 may represent an effective therapeutic strategy for the treatment of HCC.

## 1. Introduction

Liver cancer is the sixth most commonly diagnosed cancer and the third leading cause of cancer death worldwide, with an estimated 905,700 new cases and 830,200 deaths in 2020 [1]. Hepatocellular carcinoma (HCC) accounts for more than 80% of primary liver cancers globally. Approximately half of the world's HCC cases and deaths occur in China, where people have the highest burdens of HCC attributable to aflatoxin exposure and HBV infection [2]. Most early-stage HCC can be cured through surgical resection, local ablation, or liver transplantation, while multiple kinases and immune checkpoint inhibitors (such as sorafenib, lenvatinib, and nivolumab) have been effectively used in patients with advanced-stage HCC [3]. Although recent advances in HCC treatment have remarkably improved the overall survival of patients with HCC, intrahepatic recurrence, and distant metastasis are the major causes of management failure in HCC [4, 5]. Therefore, it is urgent to discover available biomarkers to predict the malignant progression of HCC and the prognosis of HCC patients.

DEAD-box helicase 21 (DDX21), a conserved Asp-Glu-Ala-Asp (DEAD) box RNA helicase, catalyzes the unwinding and separation of double-stranded RNAs and the remodeling of G-quadruplex and RNA–protein complexes [6]. DDX21 is involved in various cellular processes, including RNA polymerase II-mediated transcription, RNA splicing, and ribosome assembly [7, 8]. DDX21 modulates lymphatic vessel development in a cell-autonomous manner and is indispensable for Vegfc-Flt4-driven endothelial cell growth [9]. Moreover, DDX21 promoted cell proliferation through upregulation of cyclin D1 and CDK2 in gastric cancer [10]. DDX21 has been demonstrated to induce tumorigenesis of MYCN-amplified neuroblastoma cells via upregulation of CEP55 expression [11]. However, the role of DDX21 in the recurrence and prognosis of HCC patients remains unknown.

Herein, we investigated the protein expression of DDX21 in HCC tissues through immunohistochemical analysis and found elevated levels of DDX21 protein in HCC tissues compared with paracancerous tissues. Subsequently, we evaluated the correlation between DDX21 protein expression and clinicopathological characteristics as well as clinical outcome in patients with HCC. Our results showed that increased expression of DDX21 was associated with advanced BCLC stage. In addition, DDX21 was identified as an independent risk factor for predicting poor overall survival and recurrence-free survival in HCC patients. Notably, DDX21 silencing abated the growth and mobility of Hep3B cells. In summary, our data highlight the clinical significance of DDX21 in the recurrence and prognosis of HCC patients and indicate that DDX21 may be a potential target for treatment of HCC patients.

## 2. Materials and Methods

### 2.1. Tissue Microarrays

Commercial human HCC tissue microarrays were obtained from Shanghai Outdo Biotech Company (Shanghai, China). Inclusion criteria for this study were as follows: (1) patients underwent surgical resection; (2) histopathological diagnosis of HCC; (3) age >18 years; (4) no preoperative chemotherapy or radiotherapy. Exclusion criteria: (1) a previous history of cancers; (2) missing related information; (3) liver transplantation. The median of DDX21 level was used as the cutoff value to define the DDX21-high or DDX21-low subgroup. All patients provided signed informed consent, and this study was approved by the Ethics Committee of National Human Genetic Resources Sharing Service Platform (2005DKA21300) and performed in accordance with relevant guidelines and regulations.

### 2.2. Immunohistochemical Analysis

Immunohistochemistry analysis was executed as described previously [12]. Briefly, microarrays containing HCC and paracancerous tissues were deparaffinized and rehydrated with graded alcohol. Trypsin was used to achieve antigen retrieval. The microarrays were blocked with 5% horse serum at room temperature for 1 h and then incubated with primary antibodies against DDX21 (ab182156, Abcam) at 4°C overnight. The protein level of DDX21 was calculated independently by two pathologists based on an *H*-score method as described previously [13]. The staining intensity was scored as 0 (negative), 1 (weak), 2 (intermediate), and 3 (strong), respectively. The number of cells stained at each intensity was counted. The DDX21 expression was calculated according to the following formula: *H*-score = (the percentage of cells with weak staining × 1) + (the percentage of cells with intermediate staining × 2) + (the percentage of cells with strong staining × 3). The range of *H*-score was from 0 to 300.

### 2.3. Cell Culture

Hep3B cells were purchased from the National Collection of Authenticated Cell Cultures (Shanghai, China) and maintained in Dulbecco's modified eagle medium (DMEM) supplemented with 10% fetal bovine serum and 1% streptomycin/penicillin at 37°C in an atmosphere of 5% CO_2_.

### 2.4. Lentivirus Infection for Knockdown of DDX21

The shRNAs targeting DDX21 were cloned into GenePharma Supersilencing Vector. The packing and purification of recombinant lentivirus were conducted by GenePharma Co., Ltd. (Shanghai, China). To obtain the stable DDX21-deficient HCC cells, Hep3B cells were infected with recombinant lentivirus and screened for at least 2 weeks. The targeting sequences are listed below:

shNC: 5′-TCCTAAGGTTAAGTCGCCCTCG-3′;

shDDX21-1: 5′-GCATGAGGAATGGGATTGATA-3′;

shDDX21-2: 5′-CCCATATCTGAAGAAACTATT-3′.

### 2.5. RNA Extraction and Real-Time PCR

Total RNA of Hep3B cells was isolated using TRIzol reagent (Invitrogen, USA). The cDNA was reverse transcribed from 1 μg of total RNA using the iScript cDNA Synthesis Kit (Bio-Rad, USA). The real-time PCR was executed using CFX96 real-time PCR system (Bio-Rad, USA) with iQ SYBR Green Supermix (Bio-Rad, USA) as described previously [14]. The following primers were used: DDX21: 5′- TGCCATCAGGCTTTTGGATT-3′ (Forward) and 5′-GTCACAAAACCCACATTTGA-3′ (Reverse) and GAPDH: 5′-ACCACAGTCCATGCCATCAC-3′ (Forward) and 5′-TCCACCACCCTGTTGCTGTA-3′ (Reverse). The 2^*ΔΔ*Ct^ method was utilized to quantify relative gene expression.

### 2.6. Western Blotting Assay

Western blotting assay was carried out according to the standard protocol as described previously [15]. The following antibodies were used: DDX21 (ab182156, Abcam) and *β*-actin (mAbcam 8226, Abcam).

### 2.7. Cell Viability and Colony Formation Assay

Cell viability was determined by Cell Counting Kit-8 (CCK-8) assay (Dojindo, Japan) according the manufacturer's instructions. The absorbance at 450 nm was measured using a microplate reader. For colony formation assay, a total of 500 cells were seeded into a 24-well plate. Two weeks later, the cells were fixed with 4% paraformaldehyde for 10 min and stained with 0.5% crystal violet for 20 min. The number of colonies was quantified by ImageJ software.

### 2.8. Transwell Assay

The migration or invasion of Hep3B cells were determined by transwell chambers precoated with or without matrigel (Corning, USA) as described previously [16]. In brief, a total of 5 × 10^4^ indicated Hep3B cells in serum-free DMEM were loaded into the upper chamber, and the lower chamber was added to DMEM with 10% fetal bovine serum. After 24 h, the cells on the upper surface of the membrane were swabbed carefully. The chamber was fixed with 4% paraformaldehyde for 10 min and stained with 0.5% crystal violet for 20 min. The migrated or invaded cells were photographed and then counted using ImageJ software.

### 2.9. Statistical Analysis

All statistical analyses were performed by IBM SPSS Statistics 22.0 software. GraphPad Prism 8.4.3 software was used to generate graphics. Unpaired Student's *t* test and one-way ANOVA were utilized to compare the difference of means between two groups or more than two groups, respectively. Pearson's *χ*^2^ test was employed to analyze the difference in categorical data. Overall survival and recurrence-free survival were evaluated by using the Kaplan–Meier method with a log-rank test. The Cox proportional hazards regression model was used to assess the interrelationships between the outcome and variable over time. *p* values < 0.05 were considered statistically significant.

## 3. Results

### 3.1. DDX21 Protein was Upregulated in HCC Tissues

**Figure 1:**
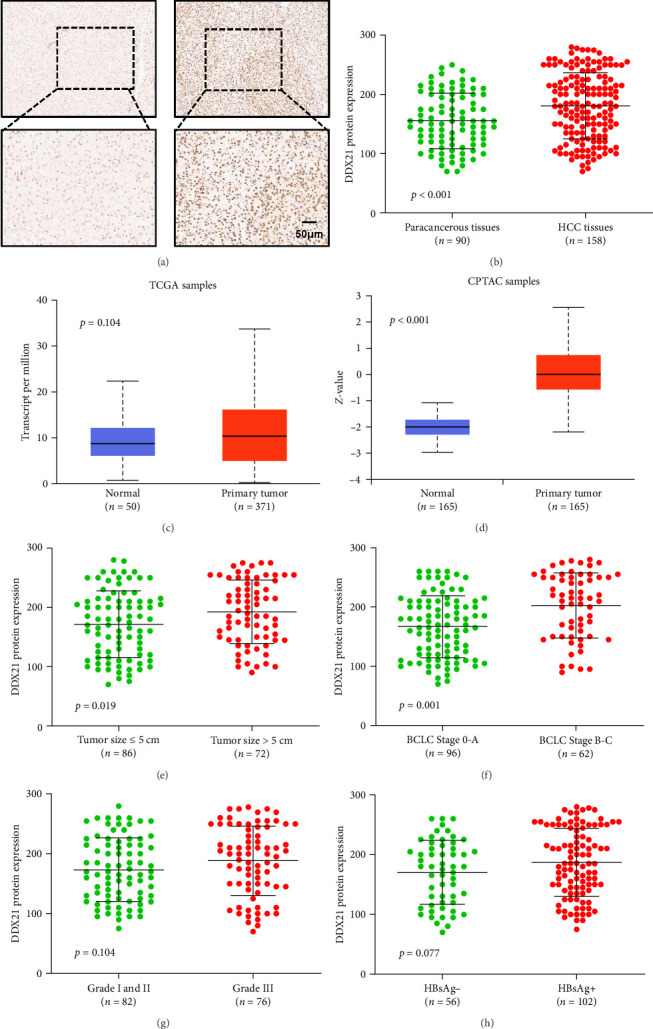
Upregulation of DEAD-box helicase 21 (DDX21) protein in hepatocellular carcinoma (HCC) tissues. (A) Representative immunohistochemical photographs of DDX21 in HCC tissues and paracancerous tissues. (B) Statistical analysis of DDX21 protein expression in HCC tissues and paracancerous tissues. (C) Analysis of DDX21B mRNA expression in normal and primary HCC tissues from the The Cancer Genome Atlas (TCGA) database. (D) Analysis of DDX21B protein expression in normal and primary HCC tissues from the Clinical Proteomic Tumor Analysis Consortium (CPTAC) database. (E) The expression of DDX21 protein in HCC subgroups classified by tumor size. (F) The expression of DDX21 protein in HCC subgroups classified by BCLC stage. (G) The expression of DDX21 protein in HCC subgroups classified by histological grade. (H) The expression of DDX21 protein in HCC subgroups classified by HBsAg status.

To explore the potential role of DDX21 in HCC tumorigenesis, we first examined the expression of DDX21 protein in microarrays containing 158 cases of HCC tissue and 90 cases of paracancerous tissues by immunohistochemistry staining. Our results showed that DDX21 protein was mainly distributed in the nucleus and that higher levels of DDX21 were observed in HCC tissues than in paracancerous tissues (Figure 1A,B). Although no significant change of DDX21 mRNA was detected between primary HCC tissues and normal tissues from The Cancer Genome Atlas (TCGA) database, DDX21 protein was increased in primary HCC tissues compared with normal tissues according to the Clinical Proteomic Tumor Analysis Consortium (CPTAC) database (Figure 1C,D). We also found upregulation of DDX21 in HCC patients with larger tumor sizes compared to HCC patients with smaller tumor sizes (Figure 1E). When tumors were classified by BCLC stage, elevated levels of DDX21 were obtained in advanced-stage (Stage B-C) compared with early-stage (Stage 0-A) patients (Figure 1F). HCC patients with grade I and II or HBsAg-negative exhibited reduced DDX21 expression compared to patients with grade III or HBsAg-positive, respectively. However, the difference was not statistically significant (Figure 1G,H). Next, we investigated the correlation of DDX21 protein expression with the clinicopathological characteristics of HCC patients. The median of DDX21 level was used to divide the HCC patients into DDX21-high and DDX21-low subgroups. Our results showed that increased expression of DDX21 was associated with advanced BCLC stage (Table 1). Although we observed a tendency for HCC patients with high AFP levels to display elevated DDX21 protein expression compared to HCC patients with low AFP levels, the difference was not statistically significant (*p*=0.058; Table 1). These data reveal that DDX21 protein is upregulated in HCC tissues and may contribute to the malignant development of HCC.

### 3.2. DDX21 Expression Predicts Recurrence and Poor Outcome in HCC Patients

**Figure 2:**
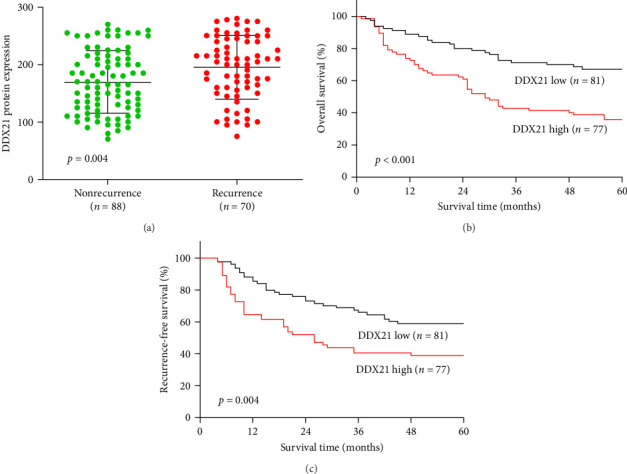
DEAD-box helicase 21 (DDX21) protein was elevated in recurrent hepatocellular carcinoma (HCC) patients and predicted poor overall survival and recurrence-free survival of HCC patients. (A) Statistical analysis of DDX21 protein expression in recurrent and nonrecurrent HCC patients. (B) The effect of DDX21 protein expression on the overall survival of HCC patients was assessed by Kaplan–Meier analysis. (C) The effect of DDX21 protein expression on the recurrence-free survival of HCC patients was assessed by Kaplan–Meier analysis. The median of DDX21 level was used to define the DDX21-high or DDX21-low subgroup.

To further clarify whether DDX21 was involved in the recurrence of HCC, we determined DDX21 protein expression in HCC tissues from HCC patients with nonrecurrence or recurrence. As shown in Figure 2A, DDX21 was considerably increased in recurrent cases compared with nonrecurrent HCC patients. Notably, the Kaplan–Meier curve showed that high expression of DDX21 was positively associated with poor overall survival of HCC patients (Figure 2B). HCC patients with high DDX21 levels had shorter recurrence-free survival than those with low DDX21 levels (Figure 2C). Univariate Cox regression analysis demonstrated that worse overall survival was correlated with high DDX21 (HR: 2.457, 95% CI: 1.522–3.965, *p* < 0.001), larger tumor size (HR: 2.284, 95% CI: 1.433–3.640, *p*=0.001), poor histological grade (HR: 1.748, 95% CI: 1.100–2.775, *p*=0.018), and advanced BCLC stage (HR: 3.560, 95% CI: 2.219–5.711, *p* < 0.001; Table 2). Shorter recurrence-free survival was associated with high DDX21 (HR: 1.961, 95% CI: 1.219–3.153, *p*=0.005), larger tumor size (HR: 1.825, 95% CI: 1.139–2.925, *p*=0.012), and advanced BCLC stage (HR: 3.676, 95% CI: 2.626–5.974, *p* < 0.001; Table 3). Multivariate analysis revealed that DDX21 expression (HR: 2.098, 95% CI: 1.294–3.403, *p*=0.003), tumor size (HR: 1.899, 95% CI: 1.184–3.047, *p*=0.008), and BCLC stage (HR: 2.958, 95% CI: 1.826–4.792, *p* < 0.001) were independent risk factors for overall survival in HCC patients. Moreover, DDX21 expression (HR: 1.734, 95% CI: 1.073–2.801, *p*=0.025) and BCLC stage (HR: 3.480, 95% CI: 2.132–5.682, *p* < 0.001) were independent risk factors for recurrence-free survival in HCC patients.

**Figure 3:**
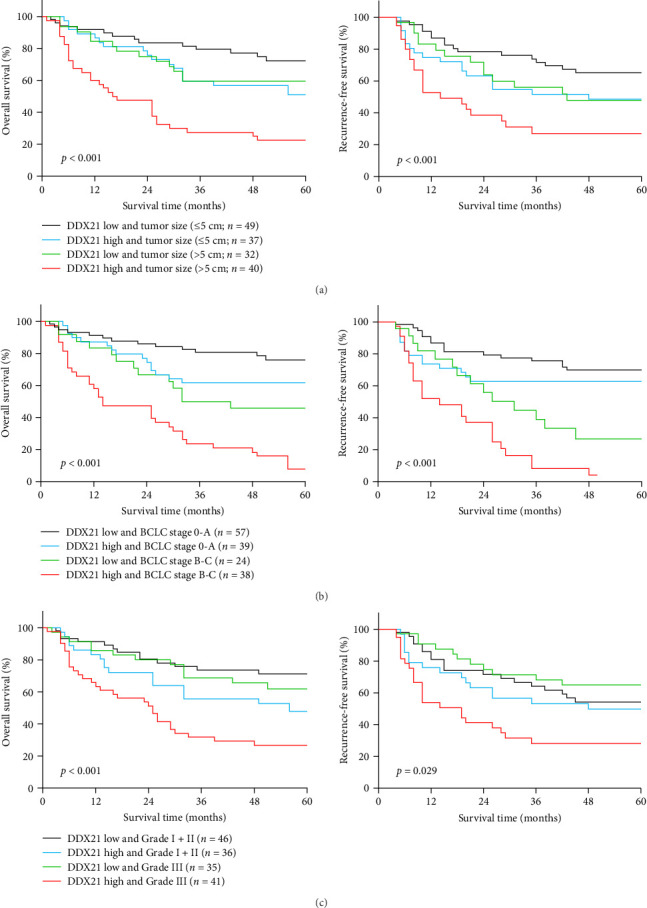
DEAD-box helicase 21 (DDX21) protein expression combined with tumor size, BCLC stage, or histological grade predicted the overall survival and recurrence-free survival of hepatocellular carcinoma (HCC) patients. (A) The effect of DDX21 protein expression in combination with tumor size on the overall survival and recurrence-free survival of HCC patients was assessed by Kaplan–Meier analysis. (B) The effect of DDX21 protein expression in combination with BCLC stage on the overall survival and recurrence-free survival of HCC patients was assessed by Kaplan–Meier analysis. (C) The effect of DDX21 protein expression in combination with histological grade on the overall survival and recurrence-free survival of HCC patients was assessed by Kaplan–Meier analysis.

According to the above results, we further investigated the combined effect of DDX21 level with tumor size, BCLC stage, and histological grade on overall survival and recurrence-free survival in patients with HCC. The subgroup of HCC patients with high DDX21 expression and larger tumor size showed worst overall survival and recurrence-free survival than the other subgroups (Figure 3A). Importantly, HCC patients diagnosed at BCLC B-C stage had considerably increased risks of mortality and recurrence, irrespective of DDX21 level (Figure 3B). In addition, elevated expression of DDX21 in combination with histological grade III predicted the shortest overall survival and recurrence-free survival in patients with HCC (Figure 3C). Our results indicate that DDX21 protein expression may act as a prospective biomarker for forecasting the overall survival and recurrence of HCC patients.

### 3.3. DDX21 Promotes the Malignant Progression in HCC

**Figure 4:**
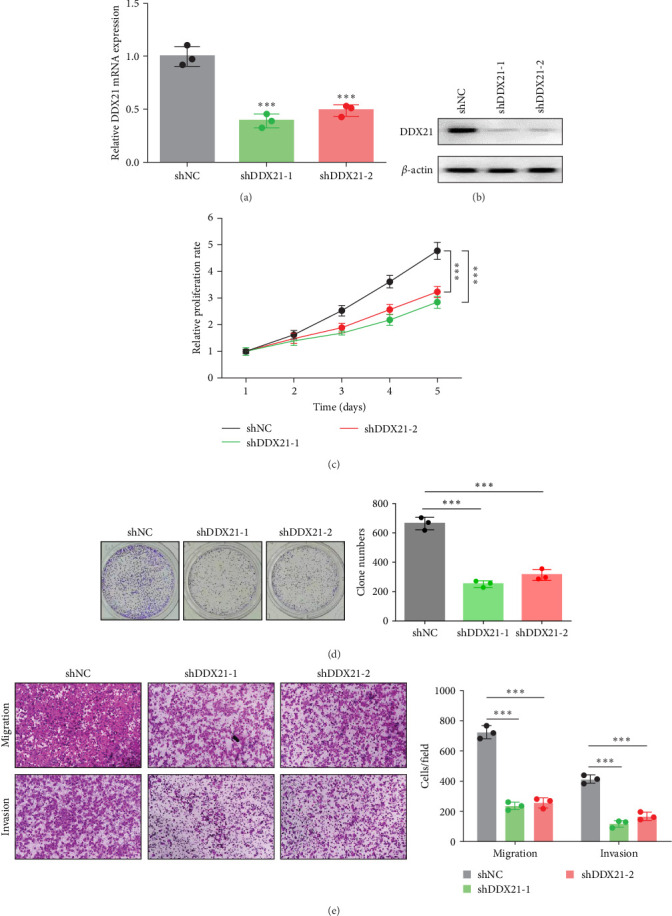
DEAD-box helicase 21 (DDX21) deficiency suppressed the growth and mobility of Hep3B cells. (A) The mRNA expression of DDX21 in Hep3B cells infected with recombinant lentivirus containing DDX21-specific shRNAs was measured by real-time PCR. (B) The protein expression of DDX21 in Hep3B cells infected with recombinant lentivirus containing DDX21-specific shRNAs was measured by western blotting. (C) The effect of DDX21 knockdown on the viability of Hep3B cells was determined by Cell Counting Kit-8 (CCK-8) assay. (D) The effect of DDX21 knockdown on the proliferation of Hep3B cells was determined by colony formation assay. (E) The effect of DDX21 knockdown on the migration and invasion of Hep3B cells was determined by transwell assay. *⁣*^*∗∗∗*^*p* < 0.001.

To examine the functional role of DDX21 on HCC progression, we manipulated DDX21 expression with two specific short hairpin RNAs (shDDX21-1 and shDDX21-2) in Hep3B cells. The interference efficiency of DDX21 was validated by real-time PCR and western blotting (Figure 4A,B). CCK-8 assay showed that DDX21 silencing considerably attenuated the viability of Hep3B cells (Figure 4C). Consistently, knockdown of DDX21 reduced the colony numbers of Hep3B cells (Figure 4D). Moreover, decreased migratory and invasive abilities were observed in DDX21-deficient Hep3B cells compared with negative control cells (Figure 4E). These data reveal that DDX21 enables to augment the proliferation, migration and invasion of HCC cells.

## 4. Discussion

DDX21 was first isolated as a nucleolar RNA helicase recognized by autoantibodies from a patient with watermelon stomach disease [17]. A previous study demonstrated a multifaceted role of DDX21 in diverse steps of ribosome biogenesis. DDX21 facilitated rRNA transcription, processing, and modification by binding to both rRNA and snoRNAs within the transcribed rDNA locus in the nucleolus. In contrast, DDX21 interacted with 7SK RNA and was recruited to the promoters of Pol II-regulated genes encoding snoRNAs and ribosomal proteins, leading to the enhanced transcription of target genes [18]. In cancer, DDX21 associated with the progesterone receptor and modulated leflunomide-induced nucleotide stress in melanoma cells [19]. Phase-separated DDX21 facilitates epithelial–mesenchymal transition (EMT) and metastasis by binding to the MCM5 gene locus in colorectal cancer [20]. Moreover, DDX21 was highly expressed in breast cancer tissues and contributed to tumorigenesis via activation of c-Jun activity and rRNA processing [21]. Paradoxically, Zhang et al. [22] unveiled that DDX21 was negatively associated with poor outcome in patients with breast cancer and acted as a metastasis suppressor through inhibition of snail-mediated EMT. These findings imply complicated function of DDX21 in regulation of different signaling pathway. In the present study, we found that the expression of DDX39B protein, but not its mRNA expression, was increased in HCC tissues compared with paracancerous tissues, suggesting posttranslational modifications of DDX21 during HCC development. In agreement with our observations, JNK phosphorylated DDX21 at serine 171, which was indispensable for maintaining DDX21 protein level and nucleolar localization [23]. Emerging evidence authenticated that ADP-ribosylated DDX21 caused by snoRNA-activated PARP-1 accelerated rDNA transcription, ribosome biogenesis, and protein translation, leading to the increased growth of breast cancer cells [24]. Long noncoding RNAs LINC00240 interacted with DDX21 and stabilized DDX21 protein by preventing USP10-mediated DDX21 ubiquitination and subsequent degradation, leading to enhanced growth and metastasis of gastric cancer cells [25]. Additionally, DDX21 expression has been modulated at posttranscriptional level. For example, miR-218-5p directly bound to the 3′-UTR of DDX21 mRNA, leading to downregulation of DDX21 in breast cancer [22]. IGF2BP2 and IGF2BP3 augmented the stability of DDX21 mRNA in an m^6^A-dependent manner, followed by recruitment of transcription factor YBX1 by DDX21 on ULK1 gene promoter and elevated transcription of ULK1, which facilitates progression of acute myeloid leukemia [26]. Therefore, an in-depth understanding of the mechanisms underlying DDX21 upregulation in HCC will be helpful for validating DDX21 as a specific biomarker of HCC.

DDX21 has been shown to induce antiviral immune responses through individual ability or formation of protein complexes in immune cells. For instance, caspase-dependent cleavage of DDX21 suppressed IFN-*β* production and host innate immunity by interrupting the assembly of the DDX1/DDX21/DHX36 complex [27]. DDX21 acted as a cytosolic sensor to recognize dsRNA and subsequently activated antiviral immune response independent of RIG-I-like receptors [28]. DDX21 promoted HIV-1 replication via enhancement of the binding between Rev and RRE. Moreover, DDX21 caused IFN-I responses and repressed dengue virus replication when DDX21 translocated from the nucleus to the cytoplasm [29]. These studies suggest an important role of DDX21 in viral replication. Here, we did not observe a relationship between DDX21 and HBsAg status. This may be attributed to the fact that DDX21 mainly senses viral RNA, while HBV is a DNA virus that is unable to be recognized by DDX21. In agreement with our results, a recent study reported that DDX21 failed to affect viral DNA replication or the formation of the viral replication compartment in human cytomegalovirus [30]. However, this study also confirmed that knockdown of DDX21 markedly inhibited human cytomegalovirus survival by promoting the accumulation of *R*-loops, which restrained RNA polymerase II elongation and prevented viral late gene transcription. These data indicate multiple roles of DDX21 in viral replication and growth. The detailed function of DDX21 in HBV-mediated HCC initiation and development needs to be further investigated in future studies.

The high recurrence rate represents one of the most vital challenges for patients after surgical resection, and the 5-year survival rate of HCC patients with recurrence is less than 40% [31]. A previous study reported that the median time from primary resection to recurrence was 22 months and the median survival from time of recurrence to death was 21 months in HCC patients [32]. However, effective biomarkers for monitoring HCC recurrence remain unresolved. In the present study, we found significantly higher DDX21 protein expression in recurrent HCC patients than in nonrecurrent cases, and elevated DDX21 protein levels were correlated with decreased recurrence-free survival in HCC patients. Multivariable Cox regression analysis revealed that DDX21 was an independent prognostic factor of overall survival and recurrence-free survival in HCC patients. Notably, the worst overall survival and recurrence-free survival were observed in HCC patients with high DDX21 expression in combination with larger tumor size, advanced BCLC stage, or poor histological grade compared with other subgroups. Consistent with our findings, a recent study reported that the expression of the DDX21 gene was increased in a variety of cancers, including brain lower grade glioma, thymoma and lymphoid neoplasm diffuse large B-cell lymphoma, and that upregulation of DDX21 was correlated with shorter overall survival in patients with adrenocortical carcinoma, endocervical adenocarcinoma, kidney renal papillary cell carcinoma, or pancreatic adenocarcinoma [7]. Thus, we infer that DDX21 may represent a valuable biomarker for predicting recurrence and clinical outcome in HCC patients.

In conclusion, our current study provides the first evidence that DDX21 protein was upregulated in HCC tissues compared with paracancerous tissues and that elevated levels of DDX21 protein were obtained in recurrent HCC patients compared with nonrecurrent cases. DDX21 expression was positively correlated with poor overall survival and recurrence-free survival in patients with HCC. Importantly, DDX21 was associated with the malignant development of HCC and was an independent risk factor for predicting the outcomes of HCC patients. Knockdown of DDX21 remarkably impaired the proliferative, migratory, and invasive abilities of HCC cells. Taken together, we propose that DDX21 may serve as a promising prognostic biomarker for HCC patients and that targeting DDX21 may represent an effective therapeutic strategy for the treatment of HCC.

## Figures and Tables

**Table 1:** The correlation between DEAD-box helicase 21 (DDX21) expression and clinicopathological characteristics in hepatocellular carcinoma (HCC) patients.

Characteristic	DDX21 expression		*p* values
	Low (*n* = 81)	High (*n* = 77)	
Age (years)	—	—	0.341
≤60	67	59	—
>60	14	18	—
Sex	—	—	0.189
Female	13	7	—
Male	68	70	—
Tumor number	—	—	0.119
Solitary	74	64	—
Multiple	7	13	—
Tumor size	—	—	0.117
≤5 cm	49	37	—
>5 cm	32	40	—
Histological grade	—	—	0.174
Well	7	2	—
Moderate	39	34	—
Poor	35	41	—
BCLC stage	—	—	0.011
0-A	57	39	—
B-C	24	38	—
Cirrhosis	—	—	0.077
No	7	14	—
Yes	74	63	—
HBsAg	—	—	0.446
Negative	31	25	—
Positive	50	52	—
AFP (ng/mL)	—	—	0.058
≤200	48	34	—
>200	33	43	—

**Table 2:** Cox regression analysis of overall survival in patients with hepatocellular carcinoma (HCC).

Characteristics	Univariate analysis			Multivariate analysis		
	HR	95% CI	*p*	HR	95% CI	*p*
DEAD-box helicase 21 (DDX21; high vs. low)	2.457	1.522–3.965	<0.001	2.098	1.294–3.403	0.003
Age (>60 vs. ≤60)	0.934	0.522–1.672	0.819	—	—	—
Sex (male vs. female)	1.175	0.564–2.448	0.667	—	—	—
Tumor number (multiple vs. solitary)	1.678	0.921–3.058	0.091	—	—	—
Tumor size (>5 cm vs. ≤5 cm)	2.284	1.433–3.640	0.001	1.899	1.184–3.047	0.008
Histological grade (III vs. I&II)	1.748	1.100–2.775	0.018	—	—	—
BCLC stage (B-C vs. 0-A)	3.560	2.219–5.711	<0.001	2.958	1.826–4.792	<0.001
Cirrhosis (yes vs. no)	1.658	0.761–3.614	0.203	—	—	—
HBsAg (Pos vs. Neg)	1.272	0.777–2.082	0.340	—	—	—
AFP (ng/mL; >200 vs. ≤200)	1.532	0.969–2.425	0.068	—	—	—

**Table 3:** Cox regression analysis of recurrence-free survival in patients with hepatocellular carcinoma (HCC).

Characteristics	Univariate analysis			Multivariate analysis		
	HR	95% CI	*p*	HR	95% CI	*p*
DEAD-box helicase 21 (DDX21; high vs. low)	1.961	1.219–3.153	0.005	1.734	1.073–2.801	0.025
Age (>60 vs. ≤60)	1.363	0.789–2.353	0.267	—	—	—
Sex (male vs. female)	1.341	0.614–2.929	0.461	—	—	—
Tumor number (multiple vs. solitary)	1.368	0.700–2.674	0.360	—	—	—
Tumor size (>5 cm vs. ≤5 cm)	1.825	1.139–2.925	0.012	—	—	—
Histological grade (III vs. I&II)	1.273	0.797–2.035	0.313	—	—	—
BCLC stage (B-C vs. 0-A)	3.676	2.626–5.974	<0.001	3.480	2.132–5.682	<0.001
Cirrhosis (yes vs. no)	0.933	0.490–1.778	0.834	—	—	—
HBsAg (Pos vs. Neg)	1.011	0.620–1.648	0.966	—	—	—
AFP (ng/mL; >200 vs. ≤200)	1.213	0.759–1.938	0.420	—	—	—

## Data Availability

The datasets used and/or analyzed during the current study are available from the corresponding author on reasonable request.
